# Examining Brain Morphometry Associated with Self-Esteem in Young Adults Using Multilevel-ROI-Features-Based Classification Method

**DOI:** 10.3389/fncom.2017.00037

**Published:** 2017-05-22

**Authors:** Bo Peng, Jieru Lu, Aditya Saxena, Zhiyong Zhou, Tao Zhang, Suhong Wang, Yakang Dai

**Affiliations:** ^1^Suzhou Institute of Biomedical Engineering and Technology, Chinese Academy of SciencesSuzhou, China; ^2^University of Chinese Academy of SciencesBeijing, China; ^3^Changchun Institute of Optics, Fine Mechanics and Physics, Chinese Academy of SciencesChangchun, China; ^4^School of Information Science and Engineering, Changzhou UniversityChangzhou, China; ^5^Trauma Center, Khandwa District HospitalKhandwa, India; ^6^Department of Neuroscience, The Third Affiliated Hospital of Soochow UniversityChangzhou, China

**Keywords:** self-esteem, magnetic resonance imaging (MRI), multilevel ROI features, brain connections, multi-kernel support vector machine

## Abstract

**Purpose:** This study is to exam self-esteem related brain morphometry on brain magnetic resonance (MR) images using multilevel-features-based classification method.

**Method:** The multilevel region of interest (ROI) features consist of two types of features: (i) ROI features, which include gray matter volume, white matter volume, cerebrospinal fluid volume, cortical thickness, and cortical surface area, and (ii) similarity features, which are based on similarity calculation of cortical thickness between ROIs. For each feature type, a hybrid feature selection method, comprising of filter-based and wrapper-based algorithms, is used to select the most discriminating features. ROI features and similarity features are integrated by using multi-kernel support vector machines (SVMs) with appropriate weighting factor.

**Results:** The classification performance is improved by using multilevel ROI features with an accuracy of 96.66%, a specificity of 96.62%, and a sensitivity of 95.67%. The most discriminating ROI features that are related to self-esteem spread over occipital lobe, frontal lobe, parietal lobe, limbic lobe, temporal lobe, and central region, mainly involving white matter and cortical thickness. The most discriminating similarity features are distributed in both the right and left hemisphere, including frontal lobe, occipital lobe, limbic lobe, parietal lobe, and central region, which conveys information of structural connections between different brain regions.

**Conclusion:** By using ROI features and similarity features to exam self-esteem related brain morphometry, this paper provides a pilot evidence that self-esteem is linked to specific ROIs and structural connections between different brain regions.

## Introduction

Self-esteem is defined as the degree that people evaluate and accept themselves (Wang and Ollendick, 2001), which has effects on human health, average lifetime, and life satisfaction (Baumeister et al., 2003). Self-esteem is concerned with a diverse array of emotions (Brown and Marshall, 2001). Low self-esteem leads to negative outcomes, such as delinquency, substance abuse, depression, and poor health condition (Baumeister et al., 2000; Marsh and Craven, 2006). Conversely, high self-esteem is associated with positive attitudes and behaviors, such as happiness, interpersonal success, ability to overcome difficulties, and healthy lifestyle (Baumeister et al., 2003). These results reveal that positive self-regard plays an important role in good emotion management and strong coping skills (Mauss et al., 2007; MacCann et al., 2011).

In recent years, self-esteem has been thoroughly researched on behavioral science. However, neuroimaging-based brain structural studies related to self-esteem are not sufficient (Heimpel et al., 2006). Among various neuroimaging techniques, magnetic resonance (MR) imaging is a secure and reliable manner to image brain structure, especially for soft tissues in the brain (Ashburner and Friston, 2000). Based on brain MR images, most researchers use volumetric or cortical analysis method to study self-esteem related brain morphometry (Agroskin et al., 2014). Pruessner et al. (2005) use volumetric analysis method to study medial temporal lobe in both young and elderly subjects, which reveals the relationship between self-esteem and hippocampal volume. Onoda et al. (2010) find out that differences in brain connections are existed between low self-esteem group and high self-esteem group. Except for these volumetric studies, cortical measurement is also used for self-esteem (Somerville et al., 2010). Beer et al. (2010) demonstrate that medial prefrontal cortex and orbitofrontal cortex are related to self-evaluation. In addition, researchers find out that self-esteem can be traced back to specific cerebral regions that involve emotional coping strategies, such as threaten, stress, anxiety, and fear. (Martyn-Nemeth et al., 2009; Cavallo et al., 2012). Detecting self-esteem related brain regions is of high value for both clinical and academic research. To our knowledge, few studies use automatic classification method to extract self-esteem related brain regions. More studies are required to further explain the relationship between self-esteem and brain morphometry.

In this paper, we examine the self-esteem related brain morphometry in regions of interest (ROIs) using multilevel-ROI-features-based classification method. Multilevel ROI features consist of ROI features and similarity features, which are extracted from T1-weighted structural brain MR images. These two types of features are complementary to each other in revealing neuroanatomical information about self-esteem. In order to reduce the dimension of features and select the most discriminating regions related to self-esteem, a mixed feature selection pattern is adopted in this study. A machine-learning-based multi-kernel support vector machine (SVM) is constructed to train the optimal classifier.

## Materials and methods

### Subjects

Characteristics of all subjects are shown in Table 1. Sixty-eight undergraduate students from Soochow University, aged from 21 to 26 years old, are included in our study. T1-weighted structural brain MR images are acquired from all subjects using a 3-T Siemens Medical Systems equipment with a standard head coil. The scanning parameters are as follows: repetition time (TR) = 2,300 ms, echo time (TE) = 2.98 ms, flip angle (FA) = 9°, field of view (FoV) = 256 mm, slice thickness = 1 mm, voxel size = 1 × 1 × 1 mm^3^. Each participant receives a structured clinical interview by a psychiatrist to rule out any psychiatric or neurological diagnoses. None of them has received stimulant or hypnagogic medication previously. All subjects have normal or corrected to normal vision and are right-handed. After the assignment, all participants are rewarded by giving small gifts or financial payments. The study is approved by the Ethics Committee of the Third Affiliated Hospital of Soochow University. Written informed consents are obtained from all subjects.

**Table 1:** **Subject characteristics**.

	**High self-esteem group**	**Low self-esteem group**	**Total**	**Statistics**	*p*-value^*^
**VARIABLES**					
No. of subjects (n)	34	34	68		
Gender (M/F)	19/15	16/18	35/33	chi-sq = 0.05	0.83
Age (years)	21.90 ± 1.16	22.53 ± 1.42	22.21 ± 1.35		
Age range (years)	21–26	21–26	21–26	*t* = −1.6	0.15
**SCALE SCORE**					
Rosenberg Scale	25.35 ± 0.81	17.86 ± 3.35	21.61 ± 3.90	*t* = 6.32	<0.001

* *p < 0.05*.

All participants have performed the Rosenberg Self-esteem Scale (RSES) test (Leary and Baumeister, 2000; Robins et al., 2001; Martin-Albo et al., 2007) with 10 items. This neuropsychological scale is widely used to measure self-esteem level. The RSES scores of all subjects are ranked from the highest to the lowest. Then, they are divided into two groups: high self-esteem group and low self-esteem group. The high self-esteem group consists of the top 50 percent of all subjects (*N* = 34), while the low self-esteem group consists of the low 50 percent (*N* = 34) of all subjects.

### Image analysis

All brain MR images are processed and analyzed using BrainLab software (Peng et al., 2015). First, the original images are reoriented and resampled to a standard format. N3 bias correction (Sled et al., 1998) is performed to eliminate the intensity inhomogeneity. Next, skull, scalp, and dura are removed from the preprocessed images using brain extraction tool (BET) (Smith, 2002) and brain surface extractor (BSE) (Shattuck et al., 2001). After brain extraction, a level-sets-based tissue segmentation algorithm (Wang et al., 2013) is used to separate gray matter (GM), white matter (WM), and cerebrospinal fluid (CSF). Then, the tissue segmented images are registered to brain atlas using an automatic method (Thirion, 1998; Wu et al., 2014). The brain atlas is based on the Automated Anatomical Labeling (AAL) template with 45 labeled ROIs for each hemisphere (Tzourio-Mazoyer et al., 2002). Finally, cortical surface is reconstructed using a deformable surface method (Li et al., 2012). Because subcortical regions are not researched, only 78 cortical ROIs (ignoring 12 subcortical regions) (Wang et al., 2014) are used in our study. It is worth noting that the brain atlas defines the outline of the anatomical region of each ROI. The anatomical region include GM, WM, and CSF. Because the tissue segmentation algorithm in our method can separate the GM, WM, and CSF, thus, we can compute the GM volume, WM volume, and CSF volume for each ROI. Cortical thickness and cortical surface area are also measured for each ROI.

### Feature extraction

**Figure 1:**
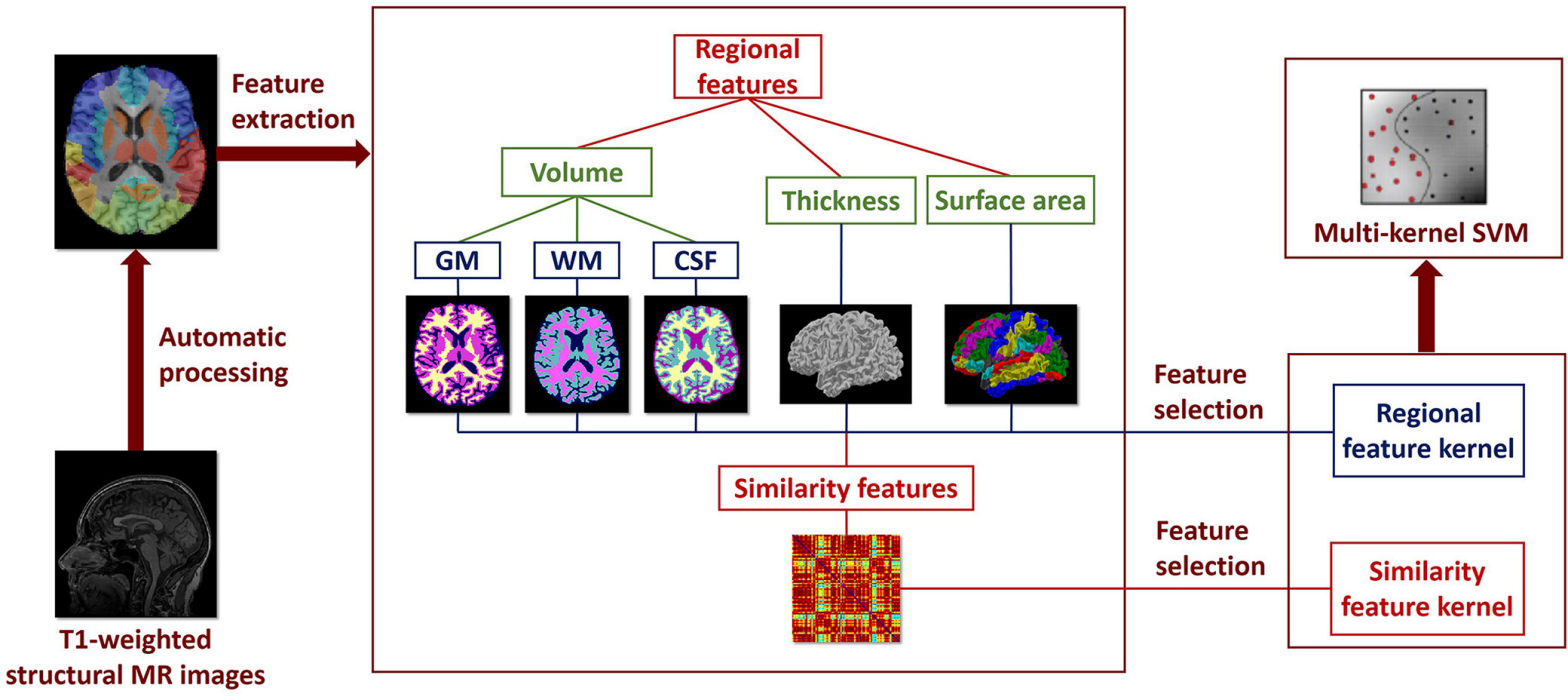
**Framework of the classification method using multilevel ROI features on T1-weighted brain MR images**.

The framework of the classification method used in our study is shown in Figure 1, including feature extraction, feature selection, and classifier construction. Two types of features are extracted from the brain MR images: ROI features and similarity features. Filter-based and wrapper-based feature selection method is applied to select the most discriminating features for each feature type, respectively. Individual kernel matrix is constructed for each feature type. Then, the individual kernel matrixes are integrated into a multi-kernel matrix. The multi-kernel matrix is used to train the optimal SVM model. Details of the method for each step will be introduced below.

The multilevel ROI features consist of ROI features and similarity features. The ROI features are automatically extracted from the brain MR images using BrainLab software, including GM volume, WM volume, CSF volume, cortical thickness, and cortical surface area. In order to decrease individual differences, the GM volume, WM volume, and CSF volume of each ROI are normalized according to the total intracranial volume of each subject (Whitwell et al., 2001), and the cortical thickness and cortical surface area of each ROI are normalized according to the standard deviation and the total cortical surface area of each subject, respectively.

Similarity features are computed based on the similarity calculation of cortical thickness between ROIs. The similarity features describe interregional information between ROIs instead of morphological information in isolated ROI, which convey high order information of brain connectivity. The integration of similarity features with ROI features will provide complementary information of the brain structure, which will improve the classification performance. In this study, a 78 × 78 similarity map was obtained for each subject. Each element in the similarity map represents the similarity value of cortical thicknesses between ROIs. Specifically, the similarity between the *i*-th and *j*-th ROIs is defined as.

(1) $$s\left(i,j\right)=exp\left\{-\frac{{\left[t\left(i\right)-t\left(j\right)\right]}^{2}}{2\sigma^{2}}\right\}\;$$

where *t*(*i*) and *t*(*j*) represent the cortical thickness values for the *i*-th and *j*-th ROIs. σ is defined as $\sigma =\sqrt{{\sigma_{i}}^{2}+{\sigma_{j}}^{2}}$ with σ_*i*_ and σ_*j*_ representing the standard deviation of cortical thickness for the *i*-th and *j*-th ROIs. Owing to the symmetrical property of the similarity map, only the upper triangular elements of the matrix are adopted to construct the feature vector. For each subject, 3003 elements in the upper triangular part are concatenated to compose a long feature vector.

The classification is performed using different feature types, including GM volume, WM volume, CSF volume, union of the above three volumes, cortical thickness, cortical surface area, similarity features, and the multilevel ROI features. The union of GM volume, WM volume, and CSF volume are constructed by a junction of the three volumes into a long vector.

### Filter-based and wrapper-based feature selection method

In order to reduce feature dimension and select the most discriminating features, filter-based and wrapper-based feature selection method is adopted. Specifically, two kinds of filter-based methods are used, followed by a wrapper-based method. First, statistical *t*-test is used to select the features that their *p*-values are smaller than the threshold (*p* < 0.05). Then, the dimension of the retained features is further reduced by the minimum redundancy and maximum relevance (mRMR) method (Ding and Peng, 2005; Peng et al., 2005). After filter-based feature selection, SVM-Recursive Feature Elimination (SVM-RFE) method (Guyon et al., 2004; Duan et al., 2005) is used to reduce the feature dimensionality. After the whole feature selection steps, we obtain the optimal feature subset for each feature type, respectively.

### Multi-kernel SVM classifier

A multi-kernel machine learning framework is adopted to integrate two types of features into a single classifier. Specifically, we first construct a kernel matrix for each feature type, respectively using a Gaussian radial basic function (RBF) kernel function. We compare the classification performance using linear kernel function and RBF function (nonlinear), which shows that the RBF kernel can significantly improve the classification performance. Thus, we choose RBF kernel function to construct the kernel matrix for each feature type. Second, these two kernel matrixes are integrated into a multi-kernel matrix with appropriate weighting factor (Wee et al., 2013). The constructed multi-kernel matrix is employed to train the optimal SVM model.

A nested crossvalidation method is applied in our study (Galar et al., 2011; Wee et al., 2012a,b). In the inner crossvalidation loop, a 2-fold crossvalidation is performed to determine the parameters of the classifier using the training set. In the outer crossvalidation loop, the generalizability of the classifier is evaluated using the testing set, which repeats 100 times. In the beginning of the experiment, the whole dataset is distributed into two parts randomly with similar number of subjects for each class in each part, one for training and the other for testing. It is worth noting that the same training and testing procedures are repeated by exchanging the training and testing sets. Paired *t*-test is used to evaluate the mean classification accuracy using multilevel ROI features compared with using other feature types.

## Results

### Classification performance

**Figure 2:**
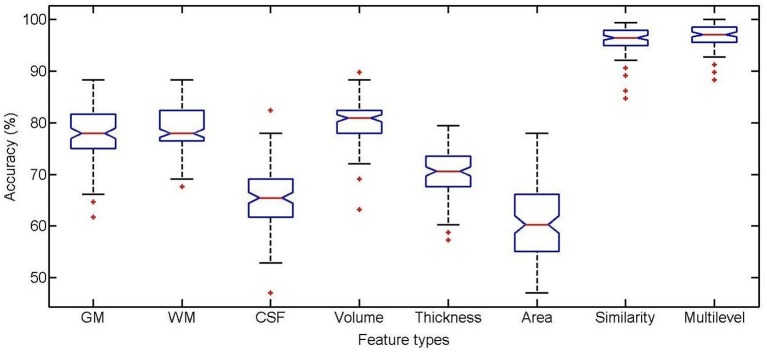
**Boxplot of classification accuracy for different feature types**.

The classification performance using different feature types between high self-esteem group and low self-esteem group is listed in Table 2, including classification accuracy (ACC), sensitivity (SEN), specificity (SPE), area under receiver operating characteristic curve (AUC), F-score (F), Youden's index (Y), balanced accuracy (BAC), and paired *t*-test results on classification accuracy. The boxplot of the classification accuracy for all feature types are showed in Figure 2. In the comparison of different feature types, the cortical surface area performs the worst in all feature types. It is found that the WM volume performs observably better than any other volumetric features (i.e., GM volume and CSF volume) with a comparatively higher classification accuracy of 86.31%. The performance of the similarity features is significantly improved comparing with using the cortical thickness. The multilevel ROI features perform the best with a classification accuracy of 96.66%, which indicates that the multilevel features possess superiority in characterizing brain morphometry between high self-esteem group and low self-esteem group. The AUC value of the multilevel ROI features is also larger than that of other feature types. Furthermore, the multilevel ROI features exhibit much better recognition ability between high self-esteem group and low self-esteem group with relatively higher specificity and sensitivity.

**Table 2:** **Mean value and standard deviation of the classification performance using different feature types**.

	**GM**	**WM**	**CSF**	**GM + WM + CSF**	**Thickness**	**Area**	**Similarity**	**Multilevel**
ACC	83.5882 (4.9001)	86.3088 (4.1182)	69.5882 (6.0336)	88.6912 (4.4496)	68.2500 (4.1176)	76.4118 (7.8679)	95.2353 (2.7921)	96.6618 (2.3262)
AUC	0.9193 (0.0455)	0.9427 (0.0359)	0.7665 (0.0630)	0.9663 (0.0301)	0.7457 (0.0382)	0.8241 (0.9334)	0.9893 (0.0019)	0.9977 (0.0027)
SEN	0.8151 (0.0490)	0.8532 (0.0412)	0.6853 (0.0603)	0.8772 (0.0445)	0.6726 (0.0412)	0.7542 (0.0787)	0.9424 (0.0279)	0.9567 (0.0233)
SPE	0.8391 (0.0781)	0.8429 (0.0619)	0.6594 (0.1266)	0.9065 (0.0884)	0.6665 (0.0890)	0.7041 (0.0996)	0.9641 (0.0391)	0.9662 (0.0356)
Y	0.8327 (0.0681)	0.8832 (0.0693)	0.7324 (0.0940)	0.8674 (0.0629)	0.6985 (0.0692)	0.8241 (0.1307)	0.9506 (0.0469)	0.9671 (0.0340)
F	0.6718 (0.0980)	0.7262 (0.0824)	0.3918 (0.1207)	0.7738 (0.0890)	0.3650 (0.0824)	0.5282 (0.1574)	0.9247 (0.0558)	0.9332 (0.0465)
BAC	0.8349 (0.0524)	0.8574 (0.0459)	0.6777 (0.0823)	0.8877 (0.0562)	0.6670 (0.0528)	0.7464 (0.0765)	0.9529 (0.0281)	0.9665 (0.0237)
*p*-value	<0.001	<0.001	<0.001	<0.001	<0.001	<0.001	<0.001	–

*GM, gray matter volume; WM, white matter volume; CSF, cerebrospinal volume; GM+WM+CSF, union of gray matter volume, white matter volume, and cerebrospinal volume; Thickness, cortical thickness; Area, cortical surface area; Similarity, similarity feature of cortical thickness; Multilevel, integration of gray matter volume, white matter volume, cerebrospinal volume, cortical thickness, cortical surface area, and similarity feature; ACC, accuracy; AUC, area under receiver operating characteristic curve; SEN, sensitivity; SPE, specificity; Y, Youden's index; F, F-score; BAC, Balanced accuracy*.

### Experiment on weighting factor

The weighting factor determines the proportion of ROI features and similarity features in the classification method. A larger weighting factor indicates that the weight of ROI features is high, which means that ROI features contribute more to the classification than the similarity features. In experiment on weighting factor, we intend to seek for an appropriate value for the weighting factor that makes the classification performance the best.

**Figure 3:**
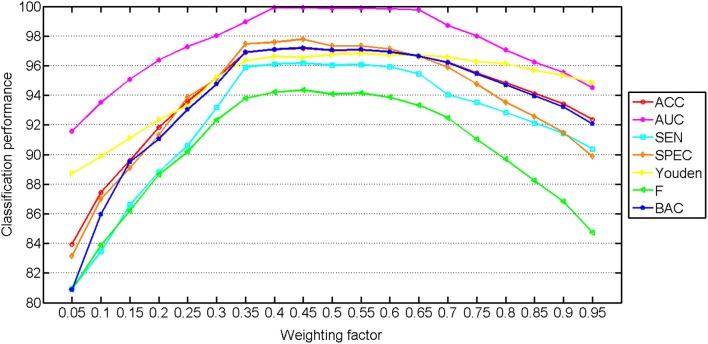
**Classification performance with multilevel ROI features using different weighting factors**. The weight for the ROI features increases from left to right (range from 0 to 1).

The classification performance with different weighting factors is shown in Figure 3. The classification performance with multilevel features using different weighting factors is performed using the whole classification method, including both training and testing. The weighting factor has significant influences on the performance of the classification. Stable and good classification performance is achieved in the range from 0.35 to 0.65, which reflects the robustness of our method. The best results were obtained at 0.65, which indicates that the ROI features and the similarity features contribute almost the same in the classification method. The wide range of the weighting factor reduces the difficulty to decide the proportion of the two feature types.

### The most discriminating features

**Figure 4:**
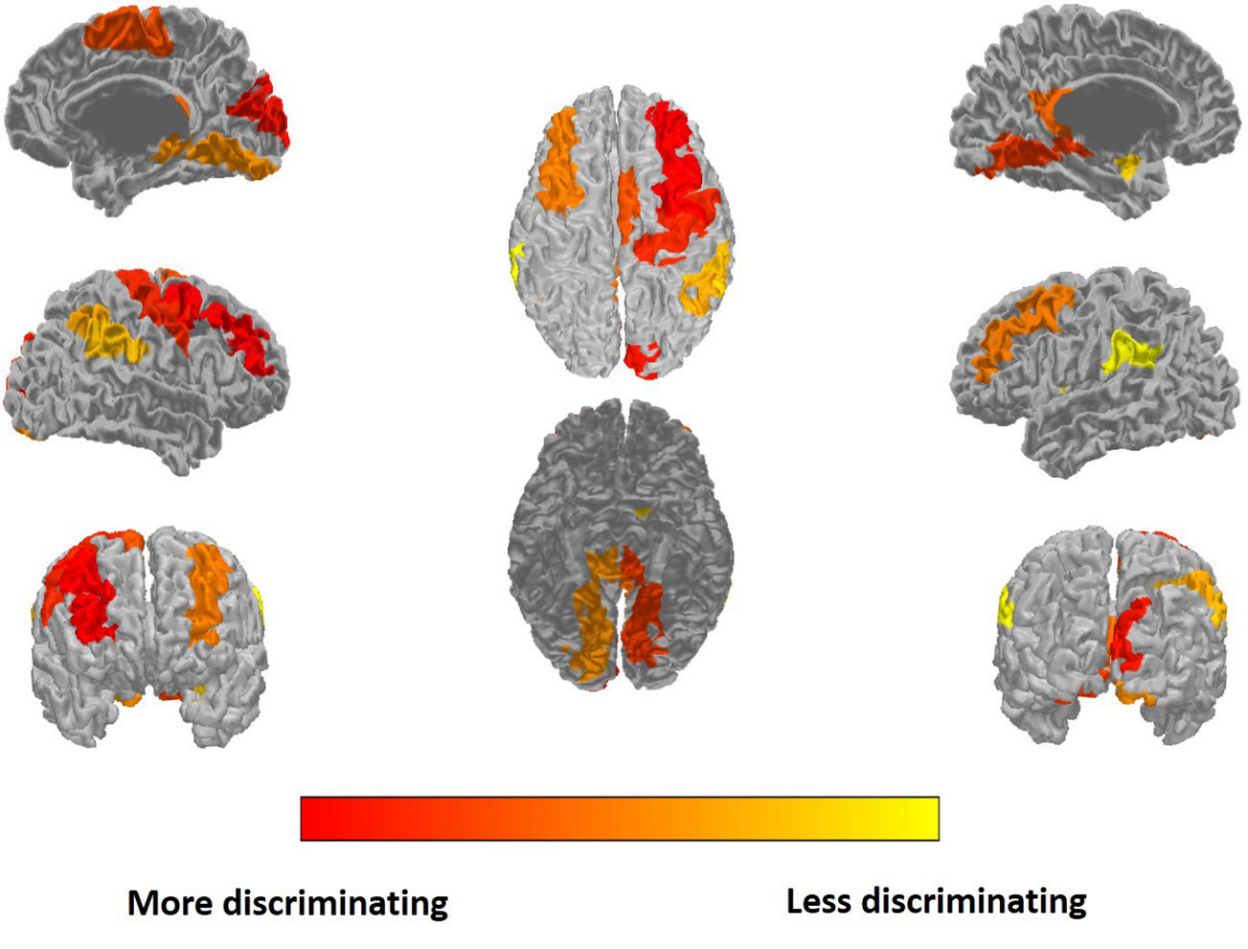
**The most discriminating ROI features projected onto the cortical surface**.

The most discriminating features are selected for ROI features and similarity features, respectively. The top 15 of the discriminating ROI features and similarity feature are listed in Table 3. The discriminating ROI features include GM volume, WM volume, CSF volume, cortical thickness, and cortical surface area. The discriminating ROI features are mainly distributed in occipital lobe (left cuneus, right superior occipital gyrus, left middle occipital gyrus, right middle occipital gyrus), frontal lobe (left middle frontal gyrus, right middle frontal gyrus, and right supplementary motor area), parietal lobe (right angular gyrus, left precuneus, right precuneus), limbic lobe (left posterior cingulate gyrus), temporal lobe (left middle temporal gyrus), and central region (right precentral gyrus). The most selected ROI features are WM volume and cortical thickness, which means that the high self-esteem group and the low self-esteem group have more brain structural differences in WM and cortical thickness than in other regions of the brain. Figure 4 shows the results of projecting the most discriminating ROI features onto the cortical surface.

**Table 3:** **Top 15 most discriminating ROI features and similarity features that were selected using the proposed classification framework**.

**No**.	**ROI features**	**Frequency**	**Similarity features**	**Frequency**
1	Middle frontal gyrus_R_W	185	Anterior cingulate gyrus_L-Middle occipital gyrus_L	95
2	Superior occipital gyrus_R_G	144	Middle frontal gyrus_R-Inferior frontal gyrus (triangular)_R	93
3	Precentral gyrus_R_T	141	Middle frontal gyrus_L-Middle occipital gyrus_L	92
4	Middle occipital gyrus_L_G	102	Middle occipital gyrus_L-Fusiform gyrus_L	85
5	Supplementary motor area_R_W	86	Middle frontal gyrus_R-Superior occipital gyrus_R	83
6	Posterior cingulate gyrus_L_C	75	Orbitofrontal cortex (superior)_L-Superior frontal gyrus (medial)_L	74
7	Middle frontal gyrus_L_W	73	Precentral gyrus_L-Inferior frontal gyrus (opercular)_L	73
8	Posterior cingulate gyrus_L_T	70	Superior frontal gyrus (dorsal)_R-Middle frontal gyrus_R	68
9	Middle occipital gyrus_R_T	68	Cuneus_L-Middle occipital gyrus_L	61
10	Angular gyrus_R_W	64	Precentral gyrus_R-Inferior frontal gyrus (opercular)_L	54
11	Precuneus_R_T	58	Middle frontal gyrus_R-Temporal pole (superior)_R	53
12	Cuneus_L_W	58	Middle frontal gyrus_R-Angular gyrus_L	48
13	Middle temporal gyrus_L_A	54	Middle frontal gyrus_L-Orbitofrontal cortex (inferior)_L	48
14	Precuneus_L_T	53	Middle frontal gyrus_R-Rectus gyrus_R	46
15	Middle occipital gyrus_L_T	53	Anterior cingulate gyrus_R-Angular gyrus_L	42

*L, left hemisphere; R, right hemisphere; G, gray matter volume; W, white matter volume; C, cerebrospinal volume; T, cortical thickness; A, cortical surface area; Frequency, selected frequency over 100 repetitions of two-fold crossvalidation*.

**Figure 5:**
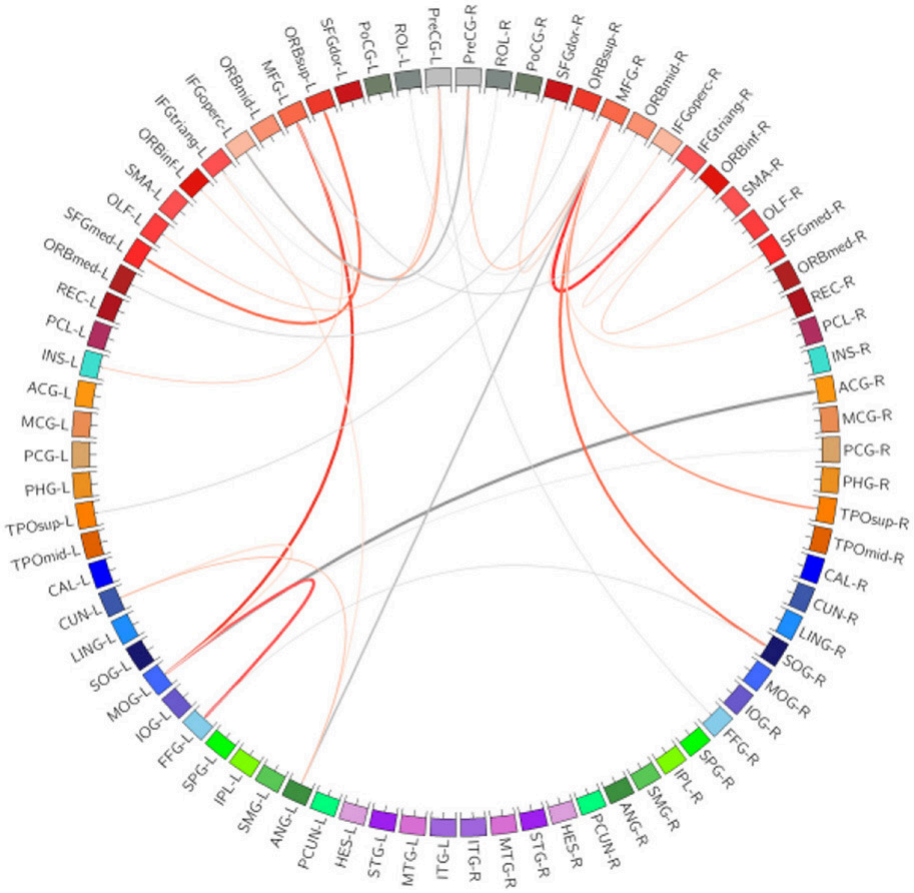
**Connection graph of the most discriminating similarity features**. Red color lines indicate relation in the same hemisphere, and gray color lines indicate relation in the two sides of the brain. Thickness of each line reflects its selection frequency, e.g., a thicker line indicates a higher selection frequency. The abbreviations of the regions can be referred to Table 4.

A connection graph of the most discriminating similarity features is shown in Figure 5, generated by Circos software (Krzywinski et al., 2009). The abbreviations of the regions can be referred to Table 4. Thicker line in the connection graph indicates stronger connection between ROIs, while thinner line implies weaker connection. The red lines represent brain connections in the same hemisphere, while the gray lines represent brain connections in different hemispheres of the brain. The most discriminating similarity features are not distributed within the same hemisphere or brain lobes, but across both the right and left side of the brain and almost across all brain regions, including frontal lobe, occipital lobe, limbic lobe, parietal lobe, and central region. Moreover, regions in the bilateral frontal lobes show closely internal relation.

**Table 4:** **Regions of interest (ROIs) defined in the automated anatomical labeling (AAL) template**.

**Index**	**Region**	**Abbreviations**	**Index**	**Region**	**Abbreviations**
1, 2	Precentral gyrus	PreCG	41, 42	Cuneus	CUN
3, 4	Superior frontal gyrus (dorsal)	SFGdor	43, 44	Lingual gyrus	LING
5, 6	Orbitofrontal cortex (superior)	ORBsup	45, 46	Superior occipital gyrus	SOG
7, 8	Middle frontal gyrus	MFG	47, 48	Middle occipital gyrus	MOG
9, 10	Orbitofrontal cortex (middle)	ORBmid	49, 50	Inferior occipital gyrus	IOG
11, 12	Inferior frontal gyrus (opercular)	IFGoperc	51, 52	Fusiform gyrus	FFG
13, 14	Inferior frontal gyrus (triangular)	IFGtriang	53, 54	Postcentral gyrus	PoCG
15, 16	Orbitofrontal cortex (inferior)	ORBinf	55, 56	Superior parietal gyrus	SPG
17, 18	Rolandic operculum	ROL	57, 58	Inferior parietal lobule	IPL
19, 20	Supplementary motor area	SMA	59, 60	Supramarginal gyrus	SMG
21, 22	Olfactory	OLF	61, 62	Angular gyrus	ANG
23, 24	Superior frontal gyrus (medial)	SFGmed	63, 64	Precuneus	PCUN
25, 26	Orbitofrontal cortex (medial)	ORBmed	65, 66	Paracentral lobule	PCL
27, 28	Rectus gyrus	REC	67, 68	Heschl gyrus	HES
29, 30	Insula	INS	69, 70	Superior temporal gyrus	STG
31, 32	Anterior cingulate gyrus	ACG	71, 72	Temporal pole (superior)	TPOsup
33, 34	Middle cingulate gyrus	MCG	73, 74	Middle temporal gyrus	MTG
35, 36	Posterior cingulate gyrus	PCG	75, 76	Temporal pole (middle)	TPOmid
37, 38	ParaHippocampal gyrus	PHG	77, 78	Inferior temporal gyrus	ITG
39, 40	Calcarine cortex	CAL			

## Discussion

Recent neuroimaging studies based on brain MR images are mainly reported on single-level morphometric measurements, such as brain volume or cortical thickness. Multilevel ROI features achieve promising classification results: accuracy = 96.66%, specificity = 99.77%, and sensitivity = 95.67%. The relatively high classification accuracy achieved in our study demonstrates that the multilevel ROI features have advantages on characterizing self-esteem related brain morphometry. In order to eliminate other factors that can interfere the experiment results, gender distribution of each group is kept balanced in our study.

The promising classification performance and the discriminating features, as reported in our study, are important for the clinical perspective, as self-esteem is a kind of complicated cognition psychology (Eisenberger et al., 2011), lacking neurological mechanism bases. Current neuroimaging studies are tried to locate specific brain regions that deal with self-cognition information. However, there are some shortcomings of these studies. Although these cognitive neuroscience studies give preliminary interpretation of relationship between information processing of self-esteem and the corresponding brain regions (Fuchs and Flugge, 2003; Gyurak et al., 2012; Zimmerman et al., 2016), network activities of specific brain areas are not taken into account in these studies. Our method can not only repair the deficiency of these existing methods but also provide information of both isolated ROI and brain connectivity between ROIs, which helps understand the development of self-evaluation and the change pattern under different self-esteem levels.

Brain regions that are related to self-esteem have been reported in previous morphometric studies. Mitchell et al. (2005) use functional MRI (fMRI) and neuropsychological test to reveal that the medial prefrontal cortex that is associated with self-reflective processing can be used to infer the mental states of other people. Morita et al. (2008) find that the right precentral gyrus plays a crucial role in self-face recognition using fMRI, which is regarded as the prerequisite for self-evaluation. Lieberman (2010) demonstrates that self-esteem correlates positively with several precuneus regions that participate in mental activity, self-referential thought, and reward. Oikawa et al. (2012) conduct an fMRI study about contrast effect in differential self-face evaluation, which shows that the posterior cingulate cortex is positively correlated with self-esteem. Frewen et al. (2013) use Visual-Verbal Self-Other Referential Processing Task and fMRI to study individual differences in neural bases, which implicates that the medial prefrontal cortex, cingulate, and precuneus are associated with self-esteem related social cognitive and affective neuroscience. Van der Meer et al. (2012) perform a self-reflection task and find that impaired insight is related to activation of angular gyrus during fMRI scanning. Middle temporal gyrus is associated with mentalizing about beliefs, desires, perceptions, or emotions of oneself and others (Gallagher and Frith, 2003; Northoff et al., 2006). Because few studies about the automatic classification of self-esteem are reported, we just compare the discriminating brain regions in our findings with existing self-esteem related morphometric studies. Consistent with these previous morphometric researches, middle frontal gyrus, precentral gyrus, posterior cingulate, angular, precuneus, cuneus, and middle temporal gyrus have also been examined in our results, which suggests the effectiveness of our classification method in revealing self-esteem related brain regions. At the same time, superior occipital, middle occipital, and supplementary motor that have not been reported in previous self-esteem related studies have been detected in our study. Further researches are needed to exclude the false positive in our results.

The most discriminating similarity features that are selected in our study are distributed in both the right and left hemisphere. Fossati et al. (2004) find that the right hemisphere has significant effects on encoding negative words under self-perception evaluation, involving right premotor cortex, right medial prefrontal cortex (dorsal), and right extra-striate cortex, which indicates that negative-evaluation is associated with right hemisphere. McKay et al. (2010) conduct an in-depth research on neural representation process of positive self-evaluation using dichotic listening method, which verifies that left hemisphere plays a dominant role in positive self-statements. These findings show that the right and left hemispheres have different self-evaluation neural representations. Positive self-statement is mainly affected by the left hemisphere, and negative self-statement is influenced by the right hemisphere. Our results of the most discriminating similarity features show the structural connections between different brain regions, which helps further study the structural connectivity characteristics related to self-esteem.

Several ROIs in the frontal lobe have been selected in our method. The human frontal lobe is primarily responsible for planning, sequencing and organizing behavior for attention, moral judgment, and self-control. After conducting thorough analysis of existing studies about self-esteem related neuropsychological mechanism, we find that prefrontal regions are the important components of self-esteem neural basis, including medial prefrontal cortex and anterior cingulate. Medial prefrontal cortex, located at the front of brain, is responsible for emotion regulation. Craik et al. (1999) use positron emission tomography (PET) to find that specific areas in middle region and right front of the brain are active when people conduct evaluation on themselves or on others. Taylor and Brown (1988) show that dorsolateral prefrontal areas are strongly active when people evaluate themselves. A self-esteem related fMRI study suggests that medial prefrontal cortex has obviously been activated when people conduct self-assessment (Heatherton et al., 2006). Anterior cingulate, located at the internal surface of the frontal lobe, is an important component associated with behavior, cognition, and emotion. Moran et al. (2006) find that anterior cingulate plays a key role in processing positive information related to self-esteem. These studies indicate that there is a crucial relationship between frontal lobe and self-esteem related cognitive characteristics.

Although the classification performance is well, there are still some limitations in our study. First, despite the fact that our data amount is relatively small, as a pilot study, we still find the self-esteem related brain regions using machine learning method. Second, the image processing algorithms may affect the brain segmentation results and thus affect the measurements of the features. In this study, the segmentation algorithms are verified in published articles (Sled et al., 1998), which makes our results credible. Third, using the whole classification method to conduct experiment on weighting factor may lead to the overfitting problem on the training set, which may overestimate the performance of the classifier. In addition, we put forward some suggestions for further study of self-esteem related cognitive neuroscience. First, considering the complexity of self-esteem in psychological process, many factors, including psychological level, individual level, and social level, should be taken into account for accurate and reliable research. Second, the relationship between self-esteem and mental diseases needs strengthen research to predict and diagnose self-esteem related diseases, such as anxiety neurosis, depression, and posttraumatic stress disorder, which is meaningful for resolving clinical problems. Third, self-esteem related studies about different races and different genders are required to explore the effects of different thinking patterns, value concepts, and world outlooks of different population groups.

## Conclusion

In this study, we use the multilevel-ROI-features-based classification method to exam self-esteem related brain morphometry. T1-weighted brain MR image are used in our study. Multilevel ROI features consist of ROI features and similarity features, which is useful to locate the specific brain regions that are processing self-cognition information. Mixed feature selection methods are applied to select the most discriminating features related to self-esteem. The discriminating features that are selected in our study are consistent with existing structural studies. Moreover, our study provides an important step in revealing self-esteem related brain structure and brain connectivity, which offers a potential research direction to further study the mechanism basis of the cognitive neuroscience of self-esteem.

## Ethics statement

This study was carried out in accordance with the recommendations of the Ethics Committee of the Third Affiliated Hospital of Soochow University with written informed consent from all subjects. All subjects gave written informed consent in accordance with the Declaration of Helsinki. The protocol was approved by the Ethics Committee of the Third Affiliated Hospital of Soochow University.

## Author contributions

Writing the article: BP; Revision of the article: BP, JL, AS, ZZ, TZ, SW, and YD; Data analysis and statistical expertise: BP and SW; Data collection: JL, AS, and SW; Obtaining funding: JL, SW, and YD.

## Funding

This study is supported partly by the National 863 Program of China (2015AA020514), NSFC grants (61501452), SSTP program (ZXY201426), Jiangsu Key Research and Development programs (BE2016613, BE2016010, BE2016010-3, BE2016010-4), postdoctoral research funding of Jiangsu province (1501089C), BKLN program, Suzhou Scientific and Technologic programs (ZXY201426, SYG201606, SZS201609), and SND Medical Plan Project (2016Z010).

### Conflict of interest statement

The authors declare that the research was conducted in the absence of any commercial or financial relationships that could be construed as a potential conflict of interest.
